# microRNA-451-modulated hnRNP A1 takes a part in granulocytic differentiation regulation and acute myeloid leukemia

**DOI:** 10.18632/oncotarget.19325

**Published:** 2017-07-18

**Authors:** Li Song, Hai-Shuang Lin, Jia-Nan Gong, Hua Han, Xiao-Shuang Wang, Rui Su, Ming-Tai Chen, Chao Shen, Yan-Ni Ma, Jia Yu, Jun-Wu Zhang

**Affiliations:** ^1^ The State Key Laboratory of Medical Molecular Biology, Department of Biochemistry and Molecular Biology, Institute of Basic Medical Sciences, Chinese Academy of Medical Sciences and Peking Union Medical College, Beijing 100005, China; ^2^ Department of Obstetrics and Gynecology, Hebei General Hospital, Shijiazhuang 050051, China

**Keywords:** acute myeloid leukemia (AML), granulocytic differentiation, hnRNP A1, C/EBPα, microRNA-451

## Abstract

Myelopoiesis is under the control of a complex network containing various regulation factors. Deregulation of any important regulation factors may result in serious consequences including acute myeloid leukemia (AML). In order to find out the genes that may take a part in AML development, we analyzed data from AML cDNA microarray (GSE2191) in the NCBI data pool and noticed that heterogeneous nuclear ribonucleoprotein A1 (hnRNP A1) is abnormally over-expressed in AML patients. Then we investigated the function and mechanisms of hnRNP A1 in myeloid development. A gradually decreased hnRNP A1 expression was detected during granulocytic differentiation in ATRA-induced-NB4 and HL-60 cells and cytokines-induced hematopoietic stem and progenitor cells. By function-loss and winning experiments we demonstrated hnRNP A1's inhibition role via inhibiting expression of C/EBPα, a key regulator of granulocytic differentiation, in the granulocytic differentiation. During granulocytic differentiation the decrease of hnRNP A1 reduces inhibition on C/EBPα expression, and the increased C/EBPα promotes the differentiation. We also demonstrated that miR-451 promotes granulocytic differentiation via targeting to and down-regulating hnRNP A1, and hnRNP A1 positively regulates c-Myc expression. Summarily, our results revealed new function and mechanisms of hnRNP A1 in normal granulocytiesis and the involvement of a feed-back loop comprising c-Myc, miR-451 and hnRNP A1 in AML development.

## INTRODUCTION

Heterogeneous nuclear ribonucleoprotein A1 (hnRNP A1) is an important RNA binding protein that exerts its activities on a wide range of cellular processes [1]. Although initially recognized as a DNA binding protein [2], it regulates several aspects of mRNA biogenesis including transcription [3, 4], constitutive and alternative splicing [5, 6], nuclear export and turnover [7] and so on. Its deregulation is related to diseases especially cancers. In breast cancer [8, 9], gliomas [10], colorectal cancer [11], and lung cancer [12, 13], the expression of hnRNP A1 abnormally increased. Furthermore it can also promote tumor invasion and connect to poor prognosis in hepatocellular carcinoma [14] and is correlated with the pathogenesis and prognosis of erythroleukemia [15] and prostate cancer [16]. Meanwhile many genes regulated by hnRNP A1 exert important functions on proliferation or differentiation, such as VDR [17], K-Ras [18, 19], c-Myc [20], GM-CSF [21] etc. hnRNP A1 was also involved in apoptosis inhibition of tumor cells by regulating XIAP [22], APAF-1 [23] etc, and siRNA-mediated knockdown of hnRNP A1 induced apoptosis in cancer cells [24]. In addition, human Let-7a [25] and IL-6 [26], which play roles in the regulation of cell pluripotency maintenance and differentiation, are also regulated by hnRNP A1.

Monocyte/macrophage differentiation and granulocyte differentiation are two important branches of hematopoiesis, which are under the control of a complex network of various regulation factors, including cytokines [27], transcription factors [28], noncoding RNAs, etc. Deregulation of any important regulation factors may result in serious consequences including hematopoietic malignancy. Acute myeloid leukemia (AML) is a heterogeneous group of blood cancers characterized by clonal expansion and differentiation blockage of myeloid progenitor blast [29]. One of the most studied lineage transcription factors is CCAAT enhancer binding protein alpha (C/EBPα) that is mainly involved in normal and malignant myelopoiesis [30, 31]. The formation of active C/EBPα homo-dimers may be a prerequisite for granulopoiesis and it can induce transcription of several regulatory proteins required for subsequent granulocytic maturation [30]. The expression and activity of C/EBPα can also be modulated by other regulators [31].

MicroRNAs (miRNAs), which negatively regulate gene expression at post-transcriptional levels [32], have also been identified as crucial regulators in normal and malignant myelopoiesis [33–35]. In a previous study, we found miR-451 is abnormally down-regulated in AML patients, and miR-451 functions as a tumor suppressor via promoting apoptosis and suppressing malignant proliferation [36].

In this study, we identified an abnormal increase of hnRNP A1 mRNA level in AML patients, and demonstrated that during granulocytic differentiation the decrease of hnRNP A1 released inhibition on *C/EBPα*, which resulted in promotion of the differentiation. We also demonstrated that miR-451 promotes granulocytic differentiation via targeting *hnRNP A1* mRNA.

## RESULTS

### hnRNP A1 is abnormally up-regulated in the AML patients

**Figure 1:**
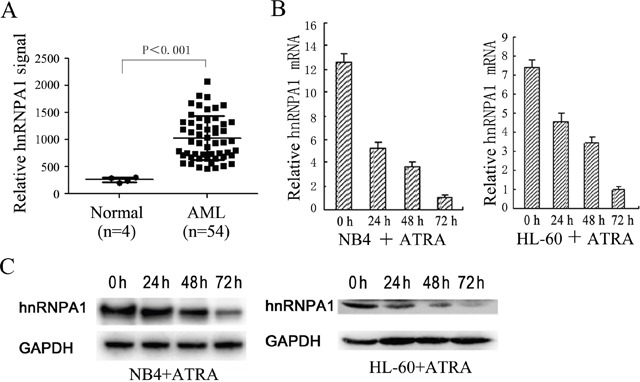
The expression levels of hnRNP A1 in AML patients and normal persons and during ATRA-induced granulocytic differentiation of NB4 and HL-60 **(A)** A comparison of hnRNP A1 mRNA levels in AML patients and normal persons. These datas come from the cDNA microarrays data from NCBI GEO database. The signal values of hnRNP A1 mRNA expression were obtained from GEO2R (online analysis software of NCBI: https://www.ncbi.nlm.nih.gov/geo/info/geo2r.html). **(B)** qRT-PCR revealed a gradual decrease in the expression of hnRNP A1 mRNA during ATRA-induced granulocytic differentiation of NB4 and HL-60. The hnRNP A1 mRNA level was normalized to GAPDH mRNA. The ralative hnRNP A1 mRNA levels are indicated as fold and the level in the cells induced by ATRA for 72 hours is normalized as 1. Error bars represent the standard deviation (SD) obtained from three independent experiments. **(C)** Western blot analysis revealed a gradual decrease in the expression of hnRNP A1 protein during ATRA-induced granulocytic differentiation of NB4 and HL-60 cells. GAPDH was used as a loading control.

We downloaded the cDNA microarrays data of AML and normal control from NCBI GEO database (Affymetrix Human Genome U95 Version 2 Array), and analyzed differentially expressed genes in bone marrow mononuclear cells (MNCs) derived from 54 AML patients and 4 healthy donors by GEO2R. The results are presented as a table of genes ordered by significance (Supplementary Table 1). From these results we noticed that hnRNP A1 is abnormally up-regulated in the AML patients compared to the health controls (Figure 1A), which suggested that hnRNP A1 might function as an oncogene in AML development.

### Gradually decreased hnRNP A1 expression was detected during all-trans-retinoic acid (ATRA)-induced granulocytic differentiation in NB4 and HL-60 cells

To investigate whether hnRNP A1 participates in myeloid differentiation, we first examined its expression during monocyte/macrophage differentiation and granulocytic differentiation in the leukemia cell lines, THP-1, NB4 and HL-60. Quantitative reverse transcription PCR (qRT-PCR) and western blot analyses revealed gradual decrease of hnRNP A1 mRNA and protein levels during ATRA-induced granulocytic differentiation of NB4 and HL-60 cells (Figure 1B and 1C). No significant hnRNP A1 expression change was found during phorbol myristate acetate-induced monocyte/macrophage differentiation of THP-1 and HL-60 cells (data not shown). These results suggested hnRNP A1 might take a part in granulocyte differentiation.

### The knockdown of hnRNP A1 promotes the ATRA-induced granulocytic differentiation of NB4 and HL-60 cells

**Figure 2:**
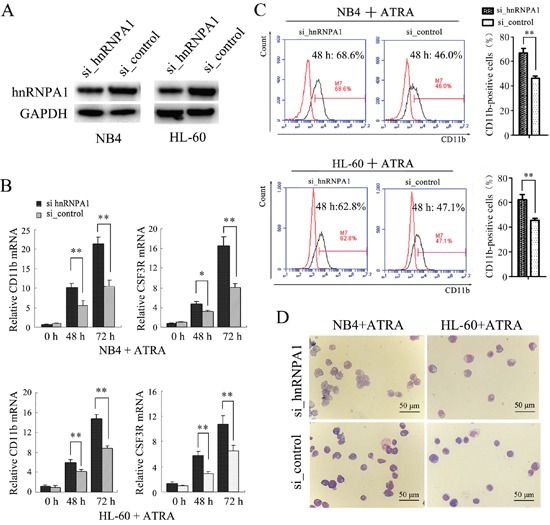
The knockdown of hnRNP A1 promotes the ATRA-induced granulocyte differentiation of NB4 and HL-60 cells **(A)** Western blot analysis of hnRNP A1 protein expression in NB4 and HL-60 cells that were transfected with hnRNP A1 siRNA (si_hnRNPA1) or siRNA control (si_control). **(B)** qRT-PCR analysis of CD11b and CSF3R mRNA expression in NB4 and HL-60 cells that were transfected with si_hnRNPA1 or si_control and subsequently treated with ATRA. The mRNA expression in uninduced si_control-transfected cell was normalized as 1. *P<0.05, **P<0.01, student's t-test. **(C)** Flow cytometry analysis of CD11b-positive cells in the transfected cells. Numbers in the graphs represent the percentages of positively stained cells after ATRA treatment for 48 hours. A representative experiment is presented in the left and a statistic analysis for three experiments in the right.**P<0.01, student's t-test. **(D)** May-Grünwald-Giemsa-staining of the NB4 and HL-60 cells that were transfected and subsequently treated with ATRA for 48 hours. Images were captured at room temperature with Olympus BX51 microscope using 20× with numeric aperture 0.5.

In order to examine the function of hnRNP A1 in granulocyte differentiation, NB4 and HL-60 cells were transfected with si_hnRNP A1 that can specifically interfere hnRNP A1 expression or si_control and then encountered ATRA induction. The knockdown of hnRNP A1 by si_hnRNPA1 transfection was confirmed (Figure 2A). qRT-PCR analysis revealed a significant increase of mRNA expression of both CD11b (a marker for both granulocytic differentiation and monocyte/macrophage differentiation) and CSF3R (a marker of granulocytic differentiation) at 48 h and 72 h ATRA induction time points in the si_hnRNPA1-transfected cells compared to the si_control-transfected cells (Figure 2B). Flow cytometry analysis revealed higher percentage of CD11b-positive cells in the si_hnRNPA1-transfected cells compared to the si_control-transfected cells (Figure 2C). May-Grünwald-Giemsa staining showed a greater fraction of more mature granulocytic cells in NB4 and HL-60 cell populations transfected with si_hnRNPA1 (Figure 2D). These results suggest that hnRNP A1 might function as a negative regulator in granulocytic differentiation.

### Enforced expression of hnRNP A1 inhibits ATRA-induced granulocytic differentiation of NB4 cells

**Figure 3:**
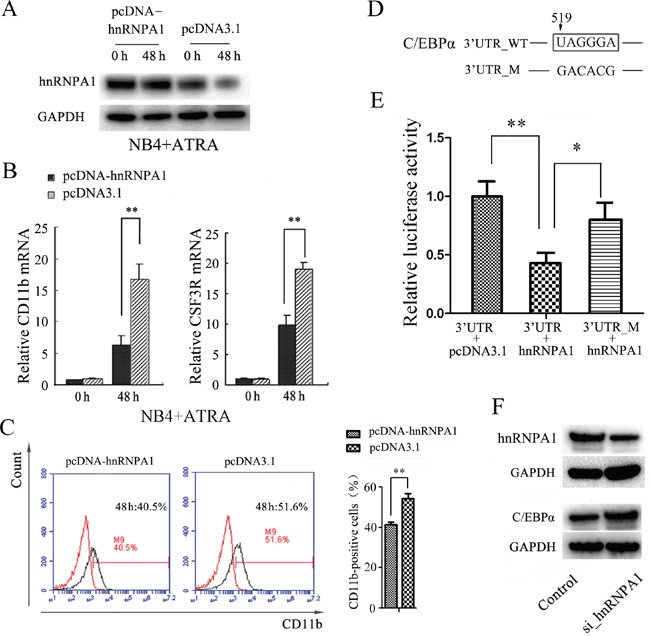
Enforced expression of hnRNP A1 inhibits ATRA-induced granulocyte differentiation and hnRNP A1 down-regulates *C/EBPα* expression in NB4 cells **(A)** Western blot analysis of hnRNP A1 protein in the NB4 cells transfected with pcDNA-hnRNP A1 or control (pcDNA3.1). The transfected cells were subsequently treated with ATRA for 48 hours. **(B)** qRT-PCR analysis of the CD11b mRNA expression in NB4 cells that were transfected with pcDNA-hnRNPA1 or control and subsequently treated with ATRA for 48 hours. The mRNA expression in the uninduced control-transfected cells was normalized as 1. **P<0.01, student's t-test. **(C)** Flow cytometry analysis of the percentage of CD11b-positive cells in NB4 cells transfected with pcDNA-hnRNPA1 or control. A representative experiment is presented in the left and a statistic analysis for three experiments in the right. **P<0.01. **(D)** The potential binding site of hnRNP A1 in 3’ UTR of C/EBPα. “519” indicates the distance to end cordon. **(E)** Luciferase activity analysis for examining the effect of hnRNP A1 on the report constructs containing the 3’ UTR_WT or 3’ UTR_M. The 293T cells were cotransfected with each luciferase reporter vector, pcDNA_hnRNPA1 or control (pcDNA3.1) and pRL-TK that was used as the internal reference. The luciferase activity was calculated as a ratio of firefly to renilla luciferase activity at 48 hour after transfection and shown as mean ± SD of three separated experiments. *P<0.05, **P<0.01, student's t-test. **(F)** Western blot analysis revealed an increased C/EBPα protein expression after knockdown of hnRNP A1 by si_hnRNPA1 transfection.

We also transfected NB4 cells with the recombinant expression plasmid pcDNA-hnRNPA1 or the control (pcDNA3.1). The over-expression of hnRNP A1 by pcDNA-hnRNPA1 transfection was confirmed (Figure 3A) After 48 hours for ATRA-treatment, qRT-PCR revealed a significant increase of CD11b and CSF3R mRNAs in the NB4 cells transfected with pcDNA-hnRNPA1 compared to the cells transfected with the control (Figure 3B). Flow cytometry analysis revealed higher percentage of CD11b-positive cells in the NB4 cells transfected with pcDNA3.1-hnRNPA1 compared to the cells transfected with the control (Figure 3C). These results further confirmed the inhibition effect of hnRNP A1 on the granulocytic differentiation.

### hnRNP A1 represses C/EBPα expression in NB4 cells

To investigate how hnRNP A1 inhibits the granulocytic differentiation, we first searched for potential genes that may be regulated by hnRNP A1. It has been reported that hnRNP A1 could specifically recognize and bind to sequence UAGGGA(U) motif to regulate mRNA splicing and other steps of post-transcriptional regulation [1, 37]. By bioinformatics analysis we identified a potential hnRNP A1 binding site that is located 519 nt downstream from the termination code in 3' UTR of C/EBPα mRNA that encodes a crucial transcription factor of granulocyte differentiation (Figure 3D). Then we amplified the DNA fragment corresponding to the 3' UTR region of C/EBPα (51bp to 743bp downstream from the termination code) and the DNA fragment containing mutations of the potential binding sequence, and inserted into dual luciferase reporter plasmid respectively. Dual luciferase reporter assay revealed that hnRNP A1 bound the 3' UTR of C/EBPα and significantly inhibited the expression of luciferase (Figure 3E). Western blot analysis demonstrated that the expression of C/EBPα protein significantly increased after knockdown of hnRNP A1 by si_hnRNPA1 in NB4 cells (Figure 3F). These results suggested that hnRNP A1 negatively regulate the expression of C/EBPα, possibly at post-transcriptional level.

### hnRNP A1 plays an important role via its regulation on C/EBPα in normal granulocytiesis

**Figure 4:**
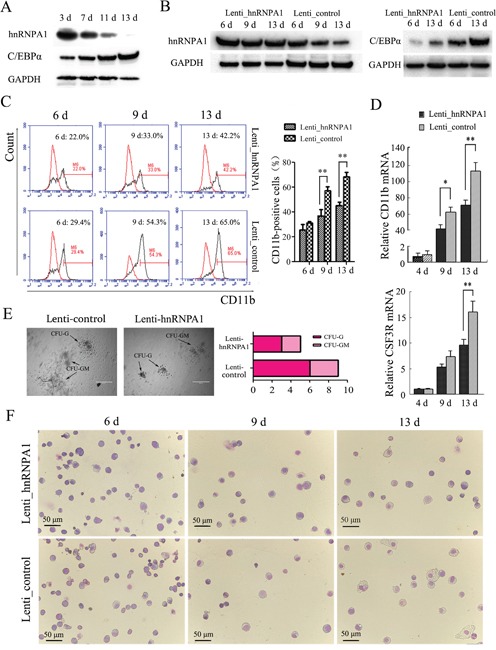
hnRNP A1 plays an important role via its regulation on C/EBPα in normal granulocytopoiesis Two sets of the experiments on CD34^+^HSPCs were performed. For each, CD34^+^ cells were purified from human UCB derived from three or four healthy donors. The CD34^+^ cells were infected with Lenti-hnRNPA1 or Lenti-control. After infection for 24 hours, the cells were cultured in a granulocytic induction medium. **(A)** Western blot analysis revealed gradually reduced hnRNP A1 expression and gradually increased C/EBP*α* expression in granulocytic induction cultures of CD34^+^HSPCs. **(B)** Western blot confirmed the ectopic expression of hnRNP A1 by Lenti_hnRNPA1 infection (left), and the ectopic expression of hnRNP A1 significantly inhibited the C/EBPα expression in the granulocytic induction cultures of HSPCs infected with Lenti_hnRNP A1 (right). **(C)** Flow cytometry analysis of CD11b-positive cells in the induction cultures of the lentiviruses-infected HSPCs. A representative experiment is presented in the left and a statistic analysis for two experiments in the right.**P<0.01, student's t-test. **(D)** qRT-PCR analysis of the differentiation marker CD11b and CSF3R mRNA expression in the induction cultures of the lentivirus-infected HSPCs (mean of experiments; n=2). The mRNA expression in day 4 induction cultures of Lenti_control-infected HSPCs was normalized as 1. *P<0.05, **P<0.01, student's t-test. **(E)** Colony formation assay. The infected CD34^+^ cells (1×10^3)^ were plated in complete methylcellulose medium without EPO. Colonies were observed at day 16 of the semisolid culture under 100 × magnification. The arrows show typical CFU-G, CFU-GM. Original bars, 400 μm. **(F)** May-Grünwald-Giemsa staining for cells in various differentiation stages at day 13 of the induction cultures. Images were captured at room temperature with Olympus BX51 microscope using 20× with numeric aperture 0.5.

To confirm the role of hnRNP A1 via its regulation on C/EBPα in the normal *granulocytiesis*, we first separated CD34^+^ cells that contain hematopoietic stem and progenitor cells (HSPCs) from human umbilical cord blood (UCB), and analyzed their expression in granulocytic induction cultures of the HSPCs. Figure 4A shows a gradual decrease of hnRNP A1 and a gradual increase of C/EBPα during the granulocyte induction cultures of the HSPCs. Then the CD34^+^HSPCs were infected with Lenti-hnRNPA1 or Lenti-control and encountered to granulocyte induction culture. The over-expression of hnRNP A1 was confirmed in the CD34^+^ cells infected with Lenti-hnRNPA1 by western blot (Figure 4B, left). Meanwhile the over-expression of hnRNP A1 resulted in a decrease of C/EBPα expression (Figure 4B, right). The differentiation status of cells was evaluated. Flow cytometry analysis revealed a lower percentage of CD11b-positive cells in the induction culture of HSPCs infected with Lenti-hnRNPA1 compared to the Lenti-control-infected cells at each indicated induction time point (Figure 4C). qRT-PCR analysis also revealed a decreased expression of CD11b and CSF3R mRNAs in the induction culture of HSPCs infected with Lenti-hnRNPA1 compared to the induction culture of HSPCs infected with lenti-control (Figure 4D). The granulocyte differentiation status was also evaluated by colony-forming assay and morphological analysis. The hnRNP A1 over-expression by lenti-hnRNP A1 infection significantly inhibited the forming of CFU-granulocyte (CFU-G) and CFU-granulocyte-macrophage (CFU-GM), reducing from both clone number and size (Figure 4E). Giemsa staining also confirmed a decrease of more mature cells in the granulocytic induction cultures of the CD34^+^ cells infected with Lenti-hnRNPA1 compared to the Lenti_control-infected CD34^+^ cells (Figure 4F). These results demonstrated that hnRNP A1 could negatively regulate granulocytic differentiation of HSPCs.

### hnRNP A1 mRNA was identified as a direct target of miR-451 in NB4 and HL-60 cells

**Figure 5:**
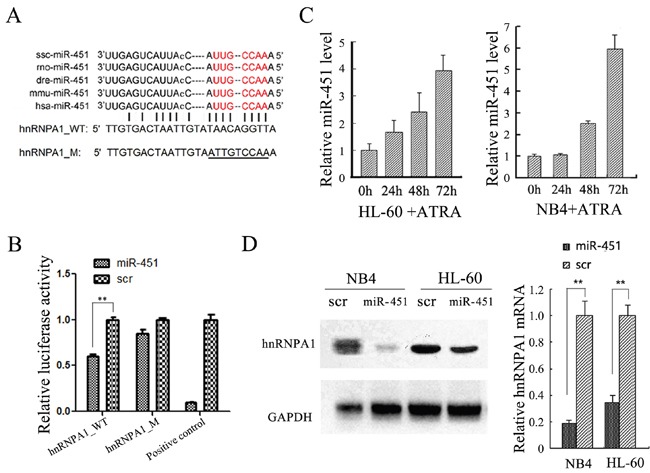
*hnRNP A1* was identified as a target gene of miR-451 in NB4 and HL-60 cells **(A)** The predicted miR-451 binding site in the 3’UTR of *hnRNP A1*. The “seed sequence” of miR-451 is marked with red color. The mutated sequence is underlined. **(B)** Enforced miR-451 expression significantly inhibited relative luciferase activity of the construct containing the wild type *hnRNP A1* 3’UTR, but not the mutant. The DNA fragment that is completely complementary to miR-451 seed sequence was used as positive control. The dual-luciferase reporter assay was performed in triplicate in HEK-293T cells. **(C)** qRT-PCR analysis revealed a gradually increased expression of miR-451 during ATRA-induced granulocytic differentiation of NB4 and HL-60 cells. **(D)** qRT-PCR and western blot analyses demonstrated that the over-expression of miR-451 by miR-451 transfection reduced *hnRNP A1* mRNA and protein levels in NB4 and HL-60 cells.

Since expression of many coding genes is post-transcriptionally regulated by miRNAs, we examined if hnRNP A1 was regulated by any miRNAs. We used TargetScan and miRanda to predict potential miRNAs targeting to hnRNP A1 mRNA and found miR-451 might target 3' UTR of hnRNP A1 mRNA (Figure 5A). We cloned the hnRNP A1> 3’UTR regions containing wild type sequence and a mutant sequence with the mutations in the seed sequence of a potential binding site into a luciferase reporter plasmid (pGL3), respectively. The dual-luciferase reporter assay in HEK-293T cells indicated that over-presence of miR-451 by miR-451 mimic transfection significantly suppresses activity of the construct with wild type *hnRNP A1* 3’UTR but not the mutant (Figure 5B). qRT-PCR analysis revealed gradually increased expression of miR-451 during ATRA-induced granulocytic differentiation of NB4 and HL-60 cells (Figure 5C), which is in contrast to the gradually reduced expression of *hnRNP A1* mRNA (see Figure 1B). Additionally, the over-presence of miR-451 by miR-451 mimic transfection also reduced hnRNP A1 expression at both mRNA and protein levels in NB4 and HL-60 cells (Figure 5D). These results demonstrated that the *hnRNP A1* mRNA was a direct target of miR-451.

### Enforced expression of miR-451 promotes ATRA-induced granulocyte differentiation of NB4 and HL-60 cells

**Figure 6:**
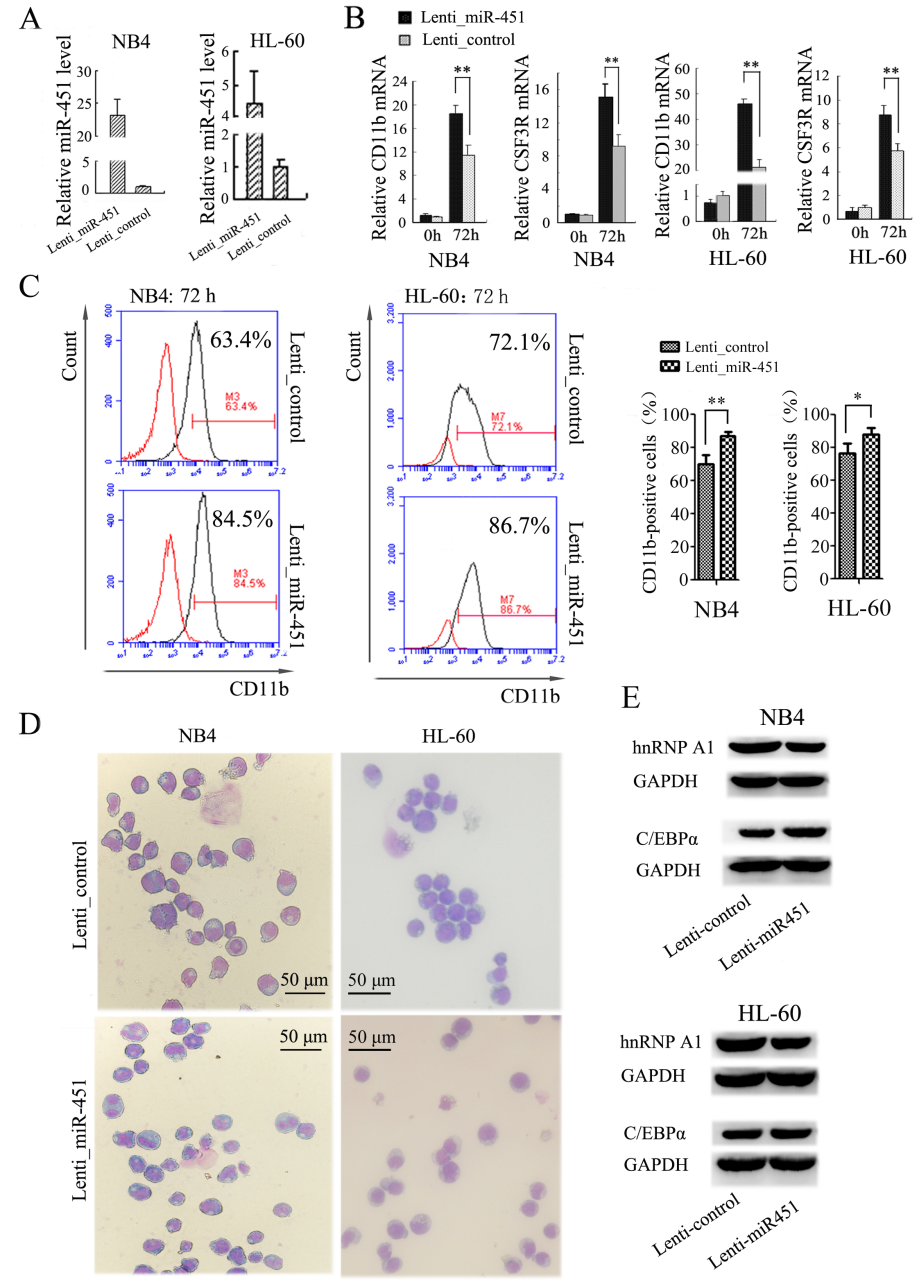
Enforced expression of miR-451 promotes the granulocyte differentiation of NB4 and HL-60 cells **(A)** Over-expression of miR-451 by Lenti-hnRNPA1 infection in NB4 and HL-60 cells was confirmed by qRT-PCR. U6 snRNA was used as an internal control. **(B)** Expression levels of CD11b and CSF3R mRNA were analyzed by qRT-PCR. qRT-PCR was performed in triplicate, and the mRNA levels were normalized to GAPDH mRNA. **P<0.01, student's t-test. **(C)** The percentage of CD11b-positive cells in the transfected NB4 and HL-60 cells was analyzed by flow cytometry analysis. A representative experiment is presented in the left and a statistic analysis for three experiments in the right.*P<0.05, **P<0.01. **(D)** May-Grünwald-Giemsa staining of NB4 and HL-60 cells that were transfected with the Lenti_miR-451 or Lenti_control and encountered ATRA induction for 48 hour. **(E)** Immunobloting analyses of hnRNP A1 and C/EBPα in the NB4 and HL-60 cells infected with Lenti_MiR-451 or Lenti-control.

To examine the functional relevance of miR-451 with granulocytic differentiation, we infected NB4 and HL-60 cells with Lenti-miR-451 or a Lenti-control. The over-expression of miR-451 by Lenti-miR-451 infection was confirmed by qRT-PCR (Figure 6A). The infected cells were encountered ATRA induction for 72 hours. qRT-PCR analysis revealed significantly increased expression of both CD11b and CSF3R mRNAs in the Lenti-miR-451-infected cells compared to the Lenti-control-infected cells after ATRA induction (Figure 6B). Flow cytometry analysis revealed higher percentage of CD11b-positive cells in the Lenti-miR-451-infected cells compared to the Lenti-control-infected cells (Figure 6C). May-Grünwald-Giemsa staining showed a significant greater fraction of more mature granulocytic cells in NB4 and HL-60 cell populations infected with Lenti-miR-451 (Figure 6D).

We also examined if miR-451 has an effect on C/EBPα expression. By bioinformatics analysis we failed to detect any potential binding site of miR-451 in C/EBPα gene. An additional dual luciferase reporter assay demonstrated no effect of miR-451 on expression of the report gene containing 3’ UTR of C/EBPα (data not shown). However, the over-expression of miR-451 by Lenti-miR-451 infection resulted in decreased hRNAP A1 but increased C/EBPα expression in NB4 and HL-60 cells (Figure 6E). These results suggested that miR-451 affect C/EBPα expression via hnRNP A1.

**Figure 7:**
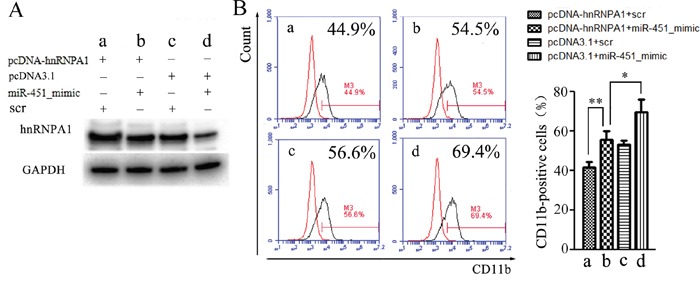
Rescue assays NB4 cells were transfected with miR-451 mimic or scramble oligonuleotides (scr) for 24 hours, re-transfected with pcDNA-hnRNPA1 or control (pcDNA3.1) for another 24 hours, and then the granulocytic differentiation was induced by ATRA for 48 hours and the cells were harvested. **(A)** The harvested cells were analyzed by western blot using the hnRNP A1 antibody. A representative immunoblotting is shown. **(B)** The harvested cells were analyzed by flow cytometry assay using the CD11b antibody. A representative experiment is presented in the left and a statistic analysis for three experiments in the right.*P<0.05, **P<0.01.

To confirm whether the miR-451 regulation of granulocytic differentiation occurred via its regulation on hnRNP A1, we performed rescue assays in NB4 cells. As shown in Figure 7, the decrease of hnRNP A1 protein expression (Figure 7A, 7Ad vs. 7Ac) was accompanied by increased CD11b-positive cells (Figure 7B, 7Bd vs. 7Bc) after miR-451 transfection. As expected, retransfection with pcDNA-hnRNPA1 reduced the decrease of hnRNP A1 expression resulting from miR-451 transfection (Figure 7A, 7Ab vs. 7Ad), which was accompanied with restoration of the percentage of CD11b-positive cells (Figure 7B, 7Bb vs. 7Bd).

Overall, our data suggested that miR-451 promotes granulocytic differentiation via its targeting and down-regulating *hnRNP A1* at least partially.

### A feed-back loop comprising c-Myc, miR-451 and hnRNP A1 is involved in AML development

**Figure 8:**
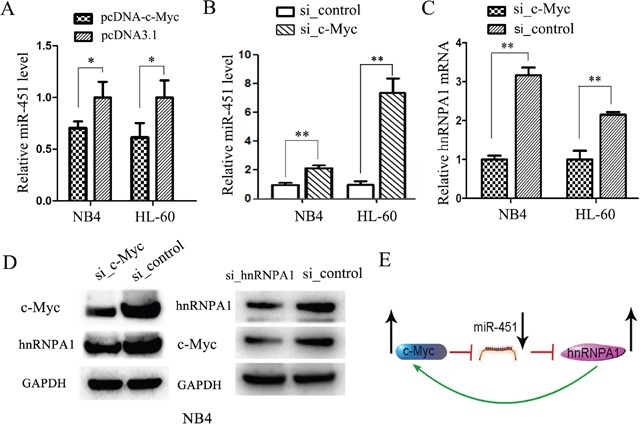
A feed-back loop comprising c-Myc, miR-451 and hnRNP A1 is involved in AML development **(A)** qRT-PCR analysis of miR-451 in the NB4 and HL-60 cells transfected with pcDNA-c-Myc or pcDNA3.1 control. qRT-PCR was performed in triplicate, and U6 snRNA was used as an internal control. *P<0.05, student's t-test. **(B)** qRT-PCR analysis of miR-451 in the NB4 and HL-60 cells transfected with si-c-Myc or si_control. qRT-PCR was performed in triplicate, and U6 snRNA was used as an internal control. **P<0.01, student's t-test. **(C)** qRT-PCR analysis demonstrated that the siRNA-mediated knockdown of *c-Myc* reduced *hnRNP A1* mRNA levels. The mRNA levels were normalized to GAPDH mRNA. **P<0.01, student's t-test. **(D)** Western blot analysis revealed that the knockdown of c-Myc by si_c-Myc transfection reduced the hnRNP A1 protein expression, while the knockdown of hnRNP A1 by si_hnRNPA1 reduced the c-Myc protein expression. GAPDH was used as a loading control. **(E)** A representative for a feed-back loop comprising c-Myc, miR-451 and hnRNP A1 is involved in AML development.

In previous studies, we found abnormally increased c-Myc expression [38] and abnormally decreased miR-451 expression [36] in AML patients, and also confirmed the miR-451 expression inhibition by c-Myc [36]. hnRNP A1 has been reported to constitutively bind to c-Myc internal ribosome entry site and mediate translation of c-Myc mRNA [39]. In the present study we demonstrated the negative regulation of hnRNP A1 by miR-451 and found abnormally over-expression of hnRNP A1 mRNA in AML patients. Thus, we examined the relation among c-Myc, miR-451 and hnRNP A1. The enforced c-Myc expression by pcDNA-c-Myc trasfection reduced miR-451 level in NB4 and HL-60 cells (Figure 8A), whiles knockdown of c-Myc by si_c-Myc transfection increased miR-451 level (Figure 8B), but reduced hnRNP A1 mRNA (Figure 8C) and protein levels (Figure 8D, left). Western blot analysis also demonstrated that the knockdown of hnRNP A1 resulted in decrease of c-Myc protein level (Figure 8D, right), suggesting a positive regulation of c-Myc by hnRNP A1 in NB4 cells. These results suggested that a feed-back loop comprising c-Myc, miR-451 and hnRNP A1 is involved in AML development (Figure 8E).

## DISCUSSION

hnRNP A1 is a multifunctional protein and plays important roles in a wide range of cellular processes [1]. In the present study we detected gradually decreased hnRNP A1 during ATRA-induced granulocytic differentiation of HL-60 and NB4 cells and during granulocytic induction culture of CD34^+^HSPCs, and by function-loss and winning experiments we observed its inhibiting role in the differentiation.

hnRNP A1 regulates almost all steps of protein gene expression, such as transcription, splicing of primary transcripts, nuclear export and turnover, mRNA translation, and post-translational moderations [1]. In the present study we demonstrated that hnRNP A1 reduces the expression of C/EBPα, the key transcriptional factor for granulocytic differentiation, possibly at post-transcriptional level although the exact mechanisms need to be confirmed. During granulocytic differentiation the decreased hnRNP A1 expression reduces the inhibition on C/EBPα expression, and the consequent increase of C/EBPα promotes the differentiation.

The relevance in diseases of hnRNP A1 is highlighted by its abnormal over-expression in various cancers [7–13]. It regulates many genes related to cell proliferation or differentiation [17–21]. Small interfering RNA-mediated reduction of hnRNP A1 induces apoptosis in human cancer cells but not in normal mortal cell lines [24], while its high expression has anti-apoptotic effects by regulating splicing of primary caspase-2 transcript [40]. And it can bind granulocyte/macrophage colony-stimulating factor (GM-CSF), an important cytokine for the granulocytic differentiation, regulating mRNA stability [41]. Thus when found abnormal over-expression of hnRNP A1 in AML patients from the AML cDNA microarray data (GSE2191) in the NCBI GEO data pool, we speculated it might take a part in AML development.

In a previous study, we verified that miR-451 is aberrantly down-regulated in AML patients, and it functions as a tumor suppressor through promoting cell apoptosis and inhibiting cell proliferation via targeting to and down-regulating YWHAZ in AML [36]. In the present study, we demonstrated miR-451's promotion effect on granulocytic differentiation via down-regulating *hnRNP A1* expression. We also found abnormally increased c-Myc expression in AML patients in a previous study [38] and hnRNP A1's positive regulation on c-Myc expression in the present study. Based on these findings we deduced that a feed-back loop comprising c-Myc, miR-451 and hnRNP A1 is involved in AML development.

In summary, our results revealed new function and mechanisms of hnRNP A1 in normal granulocytiesis and the involvement of a feed-back loop comprising c-Myc, miR-451 and hnRNP A1 in AML development.

## MATERIALS AND METHODS

### Acquisition and processing of public microarray data

The public microarray data used in this study was obtained from the National Center for Biotechnology Information (NCBI) Gene Expression Omnibus (GEO) website (http://www.ncbi.nlm.nih.gov/geo). We downloaded the cDNA microarrays data of AML and normal control uploaded by Yagi et al. [42] from NCBI GEO database (Affymetrix Human Genome U95 Version 2 Array, download address: https://www.ncbi.nlm.nih.gov/geo/query/acc.cgi?acc=GSE2191), and analyzed the genes with differential expression in bone marrow MNCs derived from 54 AML patients and 4 healthy donors by GEO2R. GEO2R is an interactive web tool that allows users to compare two or more groups of samples in a GEO Series in order to identify genes that are differentially expressed across experimental conditions.

### Cell lines, cell culture and differentiation induction

The human promyelocytic cell line NB4 and human embryonic kidney cell line HEK-293T were maintained in RPMI-1640 medium (Gibco, BRL, UK) containing 10% FCS (Gibco, BRL), 50 U/ml penicillin, 50 mg/ml streptomycin (Sigma, St. Louis, MO, USA) and 2 mmol/L glutamine at 37°C in 5% CO_2_. The human promyelocytic cell line HL-60 was cultured in IMDM medium (Gibco, BRL) supplemented with 10% FCS, 50 U/ml penicillin, and 50 mg/ml streptomycin (Sigma) at 37°C in 5% CO_2_. The lentivirus packaging cell line HEK-293TN was maintained in DMEM medium supplemented with 10% FCS, 50 U/ml penicillin, and 50 mg/ml streptomycin (Sigma). HEK-293T cell line was used for dual-luciferase reporter assay and HEK-293TN was used for packaging lentivirus; while NB4 and HL-60 cells were used to identify the function of miR-451, hnRNP A1 and C/EBPα.

For granulocytic induction, ATRA (Sigma) was added to a final concentration of 2 μM for NB4 cells and 3 μM for HL-60 cells.

### RNA isolation, reverse transcription and qRT-PCR

Total RNA was extracted from the harvested cells using Trizol reagent (Invitrogen, CA, USA) according to the manufacturer's instruction. The RNA was quantified by absorbance at 260 nm and cDNA was synthesized by M-MLV reverse transcriptase (Invitrogen) from 1μg of total RNA. Stem-poop RT primer, 5’- GTCGTATCCAGTGCAGGGTCCGAGGTATTCGCACTGGATACGACAACTCAG-3’, was used for the reverse transcription of miR-451;. The primer, 5’-AAAATATGGAACGCTTCACGAATTTG-3’, was used for the reverse transcription of U6 snRNA. Oligo (dT) 18 was used for reverse transcription of mRNA. qRT-PCR was carried out in Bio-rad IQ5 real-time PCR System (Bio-Rad, CA, USA) using SYBR Premix Ex Taq kit (Takara, Dalian, China) according to the manufacturer's instructions. For mRNAs, the data were normalized using the endogenous GAPDH control. For miRNA, U6 snRNA was used as the endogenous control. Each assay was performed in triplicate. The primer sequences for qRT-PCRs were: miR-451 forward (F), 5’-CTGGAGAAACCGTTACCATTAC-3’; miR-451 reverse (R), 5’-GTGCAGGGTCCGAGGT-3’; U6 snRNA F, 5’-CTCGCTTCGGCAGCACATATACT-3’; U6 snRNA R, 5’-ACGCTTCACGAATTTGCGTGTC-3’; hnRNPA1 F, 5'-GCCCAGTCCATCACAATCAC-3'; hnRNPA1 R, 5'-GATGCTGGCCGAGTAGGAG-3'; GAPDH F, 5’-TCAACGACCACTTTGTCAAGCTCA-3’, GAPDH R, 5’-GCTGGTGG TCCAGGGGTCTTACT-3’; CD11b F, 5’-CAGACAGGAAGTAGCAGCTCCT-3’; CD11b R, 5’-CTGGTCATGTTGATGAAGGTGCT-3’; CSFR3 F, 5’-TCAAGTTGGTGCTATGGCAAGG-3’; CSFR3 R, 5’-GCTCCCAGTCTCCACAGAATC-3’.

### Western blot

Western blot was performed as described previously [43]. The following antibodies were used. Anti-GAPDH was purchased from Proteintech Group, Inc (Chicago, IL, USA); Anti-c-Myc and Anti-IgG were purchased from Cell Signaling Technology (Boston, USA); Anti-hnRNPA1 and Anti-C/EBPα were purchased from Bioworld Technology Inc (St. Louis Park, MN, USA).

### May-Grünwald-Giemsa staining

The cells were washed twice with PBS and resuspended in serum, and then the freshly prepared and air-dried cell smears were fixed in methanol for 10 min. The slides were stained in pure May-Grünwald solution for 5 min, then washed in PBS for 5 min, and incubated in a 10% Giemsa/water solution for 30 min. The slides were then washed in PBS, air dried, and observed under optical microscopy Olympus BX51 (Olympus, Tokyo, Japan).

### Flow cytometry analysis

The harvested cells were washed twice with PBS at 4°C and resuspended in 100 μl cold PBS and then were incubated with PE-conjugated anti-CD11b (BioLegend, San Diego, CA, USA) on ice for 30 min. Finally, stained cells were washed using cold PBS and fixed in 4% paraform for further analysis on an Accuri C6 flow cytometer (BD Biosciences, San Jose, CA, USA).

### CD34^+^HSPC separation and granulocytic induction culture

Human UCB was obtained from normal full-term deliveries from Beijing Hospital. The informed consent was obtained from all of the examined subjects and the related studies and methods were approved by the Ethics Committees of the Institutional review Board of Institute of Basic Medical Sciences, Chinese Academy of Medical Sciences. MNC fractions were isolated from the blood samples by Percoll density gradient [d=1.077] (Amersham Biotech, Little Chalfont, UK). The CD34^+^ HSPCs were collected from the MNCs using a human CD34 MicroBead Kit (Miltenyi Biotec, Cologne, Germany) according to the manufacturer's recommendations. The CD34^+^ cells were cultured in IMDM (Gibco-BRL) with 30% FBS, 1% bovine serum albumin, 2 mM L-glutamine, 0.05 mM 2-mercaptoethanol, 50 U/ml penicillin, 50 μg/ml streptomycin, 50 ng/ml stem cell factor and 20 ng/ml IL-3. To induce granulocytic differentiation, 20 ng/ml G-CSF and 10 ng/ml IL-6 were added. All of these cytokines were purchased from Peprotech (Rocky Hill, NJ, USA).

### Recombination lentivirus production

A 500 bp DNA fragment containing pre-miR-451 was obtained by PCR amplification from human genome DNA with the primers: F, 5’-CGGAATTCCCCT GGCTGGGATATCATCATATA-3’, R, 5’- TTG CGGCCGCGTATCTATTCCCTCCCCTACCCC-3’. A 1464 bp DNA fragment containing the *hnRNP A1* open reading frame (ORF) was obtained by PCR amplification from healthy human cDNA of leukocyte with the primers: F, 5’-CATGTCTAAGTCAGAGTCTCCTAAAG -3’, R, 5’-AACTACACCAAGGTTTCCGAAGA-3’. The amplified fragment was inserted into downstream of CMV promoter in pCDH-MCS-T2A-copGFP-MSCV vector. The lentivirus production and infection are the same as described previously [35].

### Colony-forming assays

One thousand of the transducted cells were plated in each 35-mm tissue-culture dishes containing 3 mL Human Methylcellulose Complete Media without Epo (R&D Systems, Minneapolis, MN, USA), according to the manufacturer's instructions. After 16 days of incubation at 37°C in a 5% CO_2_ incubator, CFU-G and CFU-GM were identified and counted using phase-contrast microscopy (Eclipse TS100; Nikon, Tokyo, Japan).

### Dual luciferase reporter assay

To analyze binding between hnRNP A1 and C/EBPα 3’ UTR, a 692 bp DNA fragment containing the C/EBPα 3’ UTR region was amplified using human cDNA as template and the primers: F, 5’-GAGTGGTTTGGGGTCGCCGGATC-3’; R, 5’-GCTGACCCACGACCTAGCTTTCTGG-3’. The fragment containing the wild type and the fragment with mutations of hnRNP A1 binding site were respectively inserted into pMIR-REPORT vector (Promega, WI, USA). The amplified 1464 bp of DNA fragment including hnRNP A1 ORF, which is described above, was inserted into pcDNA3.1. These constructs as well as pRL-TK were transfected into HEK-293T cell together, using Lipofectamine 2000 (Invitrogen, CA, USA). The plasmid pRL-TK containing Renilla luciferase was used as an internal control.

To analyze miR-451 target, the 3’-UTR of human hnRNP A1 containing the wild type or mutant miR-451 binding site was inserted into pMIR-REPORT. The DNA fragment containing the mutations of the predicted seed regions in hnRNP A1 mRNA sequence was created by PCR using the primers including the mutated sequences. HEK-293T cells were co-transfected with 0.4 μg pMIR-REPOTR-hnRNPA1 construct, 0.02 μg pRL-TK control, and 5 pmol of miR-451 mimic or scrambled controls (scr). The cells were harvested 48 hours post-transfection and luciferase activity was assayed with Dual-luciferase reporter assay system (Promega) according to the manufacturer's protocol. All transfection assays were carried out in triplicate.

### Oligonucleotides and cell transfection

The miR-451 mimics, si_hnRNPA1 and their negative controls were purchased from Dharmacon (Austin, TX, USA) and transfected into the AML cells with DharmFECT1 (Dharmacon) at a final concentration of 100 nM. Six hours later, the medium was changed and cells were harvested at the indicated time points for analysis.

### Statistics

Student's t-test (two-tailed) was performed to analyze the data. P<0.05 was considered significant.

## SUPPLEMENTARY MATERIALS TABLE




