# Supplementary material for: microRNA-451-modulated hnRNP A1 takes a part in granulocytic differentiation regulation and acute myeloid leukemia
Source: Oncotarget. 2017 Jul 18;8(33):55453–66. doi: 10.18632/oncotarget.19325 (PMC5589672; doi:10.18632/oncotarget.19325)
Supplement: Supplementary file 1 [file oncotarget-08-55453-s001.pdf]

microRNA-451-modulated hnRNP A1 takes a part in granulocytic differentiation regulation and acute myeloid leukemia

SUPPLEMENTARY MATERIAL

Supplementary Table 1: Differentially expressed genes comparing AML patients and health controls

See Supplementary File 1
